# Retrospective cohort study of the impact of faecoliths on the natural history of acute appendicitis

**DOI:** 10.1186/s13017-023-00486-8

**Published:** 2023-03-14

**Authors:** Mei Sze Lee, Rachel Purcell, Andrew McCombie, Frank Frizelle, Timothy Eglinton

**Affiliations:** 1 University of Otago, Christchurch, New Zealand; 2 Department of General Surgery, Christchurch, New Zealand

**Keywords:** Faecolith, Complicated appendicitis, Preoperative CT scan, Intraoperative, Histopathology, Emergency surgery

## Abstract

**Background:**

Despite acute appendicitis is one of the most common surgical emergencies, its aetiology remains incompletely understood.

**Aim:**

This study aimed to assess the rate at which faecoliths were present in acute appendicitis treated with appendicectomy and whether their presence was associated with complicated appendicitis.

**Methods:**

All adult patients who underwent appendicectomy for acute appendicitis in a 2 years period (January 2018 and December 2019) at a single institution were retrospectively reviewed. The presence of a faecolith was identified by at least one of three methods: pre-operative CT scan, intraoperative identification, or histopathology report. Patients were grouped according to the presence or absence of a faecolith and demographics, type of appendicitis and surgical outcomes analysed. Complicated appendicitis was defined as appendicitis with perforation, gangrene and/or periappendicular abscess formation.

**Results:**

A total of 1035 appendicectomies were performed with acute appendicitis confirmed in 860 (83%), of which 314 (37%) were classified as complicated appendicitis. Three hundred thirty-nine (35%) of the appendicitis cases had faecoliths (complicated 165/314 cases; 53%; uncomplicated 128/546; 23%, *p* < 0.001). The presence of a faecolith was associated with higher complications and a subsequent longer post-operative stay.

**Conclusion:**

The rigorous methodology of this study has demonstrated a higher rate of faecolith presence in acute appendicitis than previously documented. It reinforces the association of faecoliths with a complicated disease course and the importance in prioritising emergency surgery and postoperative monitoring for complications.

## Introduction

Acute appendicitis is a common surgical emergency, affecting 17.7 million people annually worldwide. It is most prevalent in the 15–19 years age group [1]. The healthcare burden for acute appendicitis is significant, with a high mean hospital cost and consequential national annual expenditure [2]. Despite its frequency, the aetiology remains incompletely understood [3, 4].

Direct luminal obstruction is thought to be a contributor to the aetiology of acute appendicitis. Multiple studies showed that luminal obstruction-associated appendicitis may have a varied aetiology such as faecoliths, pinworms, lymphoid hyperplasia, foreign bodies, amebiasis, tuberculosis, endometriosis or tumour in patients presenting with clinical appendicitis [1, 5]. In addition to luminal obstruction, other potential primary causes of appendicitis have been implicated including viral and bacterial [6]. Invasive pathogens such as Fusobacteria were found to have a positive correlation to the severity of acute appendicitis [7]. Organisms including *Escherichia coli*, Klebsiella pneumoniae, Streptococcus spp, Enterococcus and Pseudomonas aeruginosa have all been detected by culture [8] suggesting a possible causative relationship and ongoing microbiome studies are investigating the role of microbiota dysbiosis leading to acute appendicitis [9, 10]. Seasonal variations have also been shown to affect the incidence of acute appendicitis in several studies, with the highest incidence of acute appendicitis seen in spring and summer months [11].

Previous studies including large international study [3] have demonstrated the significance of faecoliths in acute appendicitis and its associated complications. However, most of the studies utilised abdominal CT scans to assess the presence of faecoliths [12–17]. Several of these studies have correlated the size and location of faecoliths in relation to the severity of appendicitis, while other studies have assessed histopathological parameters, comparing uncomplicated and complicated appendicitis [12, 14, 18]. There is to date, however, no published study that assesses the presence of faecoliths using methodology inclusive of pre-operative imaging, intra-operative findings, and histology; the composite of which may well give a more complete appraisal of their role.

In this study, we used the above methodology to assess the rate at which faecoliths were present in acute appendicitis treated with appendicectomy, and whether the presence of faecoliths was associated with complicated appendicitis.

## Methods

This study retrospectively reviewed all appendectomies performed between January 2018 and December 2019 at Christchurch Hospital, Canterbury, New Zealand. Patients were identified using the institution’s prospectively recorded electronic database that logs all acute and elective operations.

Data were collected from an electronic clinical records system. This included age, sex, admission date, length of stay, discharge date, complications and readmissions and operative data were which was created at the time of the operation using a synoptic operating note that positively or negatively recorded the intraoperative finding of a faecolith, perforation and the degree of contamination. Histological data included the presence and severity of appendicitis, perforation and the presence of faecoliths.

A faecolith was defined as present if identified by at least one of the three possible methods (pre-operative CT scan, intraoperative or histopathology report). It was assumed absent if not identified by any of the three methods. Complicated appendicitis was defined as appendicitis with perforation, gangrene and/or periappendicular abscess formation.

All patients aged 18 years old or older who had appendicectomies were included. Appendicectomies performed in conjunction with right colonic resection, other bowel resection, gynaecological and/or pelvic operations were excluded. Appendicitis treated non-operatively was excluded, as histological confirmation of the diagnosis was not available.

Statistical analyses were undertaken using Rstudio. A Mann–Whitney *U* test was used for comparing age in appendicitis versus non-appendicitis and faecolith versus non-faecolith, while Odds Ratios with *p* values and 95% confidence intervals were calculated for all other analyses.

## Results

**Fig. 1:**
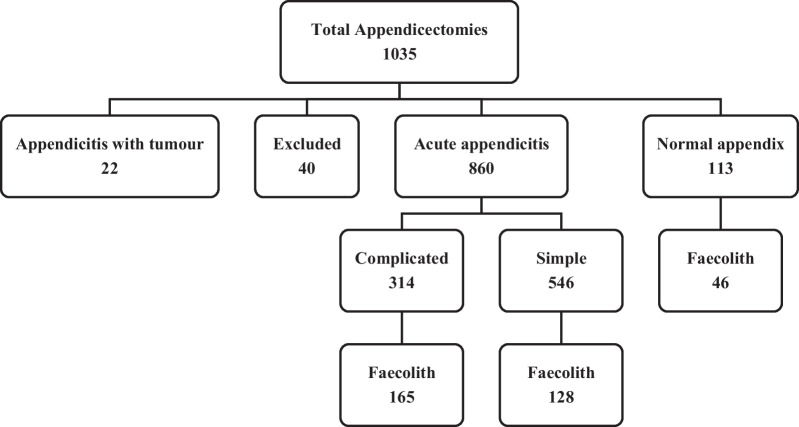
Flow diagram of all appendectomies performed

A total of 1035 appendicectomies were performed in the 2-year period. Forty cases did not fulfil the inclusion criteria, and 22 cases had acute appendicitis with a tumour. Of the remainder, 860 patients had acute appendicitis, and in 113 cases, histology demonstrated a normal appendix. Faecoliths were present in 339 of the 973 appendicectomy cases (35%) (Fig. 1).

**Fig. 2:**
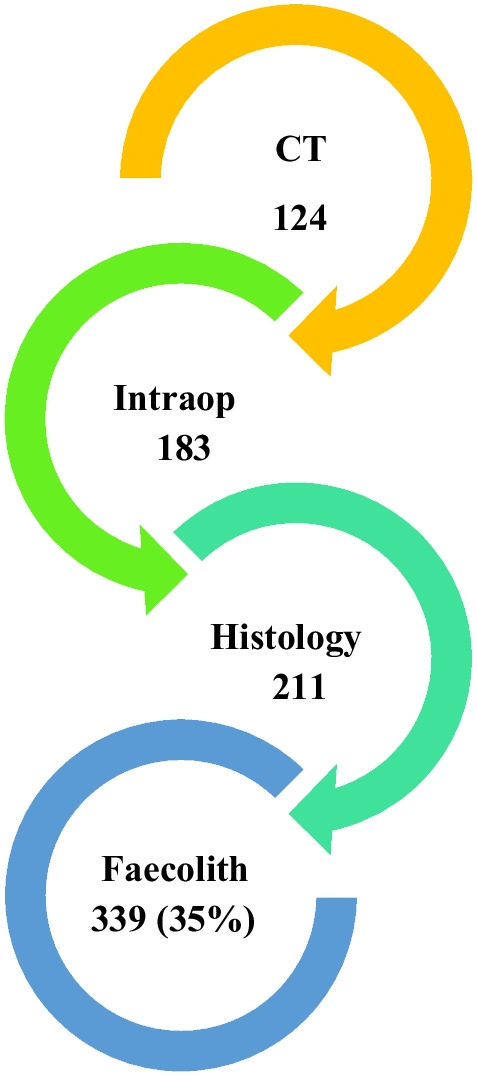
Modalities identifying faecoliths in all appendicectomies

These faecolith cases were identified using at least one of the three modalities as demonstrated in Fig. 2. The total number of faecoliths are not accumulative of each modality.

The median age of the 860 patients with histologically proven appendicitis was 35 years (IQR 25–50 years), compared to the normal appendix group median age of 23 years (IQR 20–84; *p* < 0.001). Preoperative CT scan was carried out in 423 patients (49%) in the appendicitis group and 14 cases in the normal appendix group. There was no statistically significant difference in the overall faecolith rate among patients with acute appendicitis (34%) compared to the normal appendix group (40%) (OR 0.75 and 95% CI 0.5–1.12, *p* = 0.160). (Table 1).

**Table 1:** Difference between appendicitis and normal appendix group

	Appendicitis (*n* = 860)	Normal appendix (*n* = 113)	*p* value	Odds ratio and 95% confidence interval
Median age (years)	35 (IQR 25–50)	23 (IQR 20–84)	< 0.001	
Preoperative CT scan	423	14	< 0.001	6.84 (3.85, 12.17)
Presence of faecolith(s)	293 (34%)	46 (40%)	0.16	0.75 (0.50, 1.12)
Complications	135	20	0.58	0.87 (0.52, 1.45)
Grade 1 and 2	117	19	0.38	0.79 (0.46, 1.34)
Grade 3 and 4	18	1	0.40	2.31 (0.30, 17.50)
No Complication	725	93	1 (ref)	

Among the acute appendicitis, 314 cases (37%) had complicated appendicitis (with gangrene/perforation) and 546 (64%) simple appendicitis.

The faecolith group were statistically significantly older (median 40 years (range 18–91 years)) than the non-faecolith group [median 33 years (range 18–82 years), (*p* < 0.001)]. There was no significant association between gender and the presence of a faecolith (Table 2).

**Table 2:** Demographic and clinical data for patients with acute appendicitis

Total appendicitis	860	
*Median Age (years)*	35	(IQR 25–50)
*Gender*		
Male	442	(51%)
Female	418	
*Radiology*		
CT scan	423	(49%)
No CT	239	
Other imaging	198	
*Faecolith*		
Faecolith present	293	(34%)
Faecolith not present	567	
*Severity*		
Gangrenous/Perforated	314	(36.5%)
Simple appendicitis	546	
*Median LOS (days)*	1.9	(IQR 1.4–2.8)
*Readmission*		
Yes	68	(13%)
No	592	
*Complication (CD classification)*		
Grade 1 and 2	117	
Grade 3 and above	18	
No complication	725	(82%)

A faecolith was detected in 165 of the 314 (53%) patients with complicated appendicitis compared with 128 of the 546 (23%) patients with simple appendicitis (OR 3.62, 95% CI 2.69–4.87, *p* < 0.001). After controlling for age (OR 1.03, 95% CI 1.02–1.04), the OR between faecolith and complicated appendicitis remained highly significant (OR 3.30, 95% CI 2.44–4.49). There were statistically significantly increased post-operative major complications and a longer inpatient stay in the faecolith group compared to the non-faecolith group, as demonstrated in Table 3.

**Table 3:** Difference between faecolith and non-faecolith group in the appendicitis group

	Faecolith (*n* = 293)	Non-faecolith (*n* = 567)	*p* value	Odds ratio and 95% confidence interval
Median age (years)	40 (18–91)	33 (18–82)	< 0.001	
Male	154 (53%)	288 (51%)	0.6	1.07 (0.81, 1.42)
Female	139	279		
Preoperative CT scan	174	249	0.001	1.87 (1.4, 2.49)
Simple appendicitis	128	418		
Complicated appendicitis	165	149	0.001	3.62 (2.69, 4.87)
Grade 1 and 2	46	71	0.17	1.35 (0.90, 2.02)
Grade 3 and 4	12	6	0.004	4.17 (1.55, 11.25)
None	235	490		
Readmission	25 (8.5%)	43 (7.5%)	0.65	1.14 (0.68, 1.90)
Mean LOS (days)	2.1	1.9	0.001	

## Discussion

This study demonstrated a faecolith was present in 34% of all patients with appendicitis and the presence of a faecolith was associated with a greater than threefold increase in complicated appendicitis. In previously published reports, the range of faecolith presence in acute appendicitis has varied widely from 3.6 to 17% [19, 20]. In the present study, the detection rate for faecoliths was considerably higher (34%) compared to these other published studies. This is potentially due to the more exhaustive methodology used for faecolith detection including utilising pre-operative CT scans, synoptic operating reports and histopathology reports and therefore represents a more accurate estimation of the rate of faecolith presence in acute appendicitis.

In addition to the wide reported range of rates of faecoliths in acute appendicitis, their association with the pathogenesis of appendicitis itself remains controversial. One retrospective study looked at 1357 appendicectomies and found that faecoliths were present in 13.7% of cases and showed a significant association between faecolith presence and acute appendicitis [20]. In contrast, a study by Singh et al. [21] argued that faecoliths were an incidental finding and their prevalence too low to be considered as the cause of non-perforated appendicitis. These authors analysed 1014 appendicectomies for suspected appendicitis, of which 741 were in adult and 273 in paediatric patients. They found that the overall faecolith rate was higher in normal appendices than those with acute appendicitis in both the paediatric (28.6% vs. 18.1%) and adult groups (31.6% vs 13.7%). Another large retrospective review [15] of 4670 cases showed that only 3.6% of appendicectomies contained faecoliths, in which 60.5% were faecoliths found in normal appendices.

In the current study, the rate of faecoliths present in normal appendices was 40% compared with 34% in the appendicitis group. This difference did not reach statistical significance once again challenging whether faecoliths have a role in the pathogenesis of appendicitis. Other aetiologies such as endometriosis, lymphoid hyperplasia, pin worms and carcinoid tumours were found during histology assessment in some of the appendix specimens in this study. Given the population for the study was drawn from symptomatic patients, the presence of appendiceal faecoliths may be higher than the general population if they are considered a potential cause of right iliac fossa pain in the absence of appendiceal inflammation (appendiceal colic).

Accumulating evidence suggests that appendicitis with an associated faecolith follows a more severe course than appendicitis without a faecolith. A study by Mallien et al. [14] showed a histological difference between acute appendicitis presenting with and without a faecolith. Appendicitis with a faecolith had more crypt destruction, severe acute inflammation and micro-abscesses (27.3% vs 13.4%, *p* = 0.016). A recent randomised controlled trial demonstrated an overall faecolith rate of 17% with the visualisation of faecoliths on preoperative imaging, the only factor associated with complicated appendicitis (53%; *p* < 0.0001) and with failure of antibiotic treatment (50%; *p* = 0.0072) [22]. This is further confirmed in the CODA trial where participants with appendicolith treated with antibiotics treatment were associated with higher complications (20.2 vs. 3.6 per 100 participants; rate ratio, 5.69; 95% CI 2.11–15.38) [3]. Similarly, in the present study, patients with complicated appendicitis were 3.6 times more likely to have faecoliths compared to patients with simple appendicitis and had higher rates of post-operative complications. This is also reflected in the latest WSES guideline for managing acute appendicitis where non-operative management of uncomplicated acute appendicitis is only deemed safe alternative to surgery in the absence of faecolith [4].

Complicated appendicitis is often associated with higher morbidity such as unresolved abscess or sepsis, post-operative complications, longer hospital stays and readmissions. A recent study of 150 patients who underwent appendicectomy for acute appendicitis showed that patients with perforated appendicitis were more likely to be older (64.5 years versus 38.5 years, *p* < 0.001), have an appendiceal faecolith (70%) and have longer postoperative length of stay (7 days versus 3 days, *p* < 0.001) than the non-perforated group [23]. In the present analysis, the complicated appendicitis group was statistically significantly older and four times more likely to have a moderate to severe complication. Even though the presence of faecoliths did not affect the readmission rate, there were more grade 3 complications among the readmissions within the faecolith group (12 versus 6 cases) and a prolonged inpatient stay (2.1 days versus 1.9 days, *p* 0.001). These results support the hypothesis that the presence of a faecolith in patients with acute appendicitis should be classified as high risk for complicated appendicitis warranting emergency appendicectomy and exclusion from non-operative management of acute appendicitis. The limitations of this study include its retrospective nature from a single centre, the variation in reporting of faecoliths in pathology reports and potential loss of faecoliths while transporting the specimens. Overall, this study used robust methodology which allowed a more accurate faecolith capture and rate estimation compared to other studies.

## Conclusion

The rigorous methodology of this study has demonstrated a higher rate of faecolith presences in acute appendicitis than previously documented. It reinforces that the presence of a faecolith in acute appendicitis is a strong predictor of a more severe course of acute appendicitis and thus highlights the importance of clinical prioritisation for emergency surgery and postoperative monitoring for complications.

## Data Availability

All data generated or analysed are included in this published article.
